# Clinical and biochemical footprints of inherited metabolic diseases: XVII. Dysmorphisms

**DOI:** 10.1016/j.ymgme.2024.109001

**Published:** 2024-12-13

**Authors:** Carol L. Greene, Sofia Saenz-Ayala, Erin T. Strovel, Francis Rossignol, Carlos R. Ferreira, Nenad Blau

**Affiliations:** a University of Maryland School of Medicine, Department of Pediatrics, Division of Genetics, Baltimore, MD 21201, USA; b University of Maryland School of Medicine, Department of Pathology, Baltimore, MD, USA; c NIH Undiagnosed Diseases Program, Rockville, MD, USA; d National Human Genome Research Institute, National Institutes of Health, Bethesda, MD, USA; e Division of Metabolism, University Children’s Hospital Zürich, Zurich, Switzerland

**Keywords:** Dysmorphic features, Morphology, IMD, IEM

## Abstract

Dysmorphisms, or physical abnormalities in appearance, can vary in frequency and severity among individuals with inherited metabolic disorders (IMD). The prevalence of dysmorphisms in these disorders can range from rare occurrences to more common features, depending on the specific disorder and its genetic characteristics. It is important to note that not all individuals with IMDs will exhibit dysmorphic features, and the presence of such features may vary widely among different types of metabolic disorders. The data presented in this study, which includes a detailed list of 374 IMDs with dysmorphic characteristics categorized by affected organs (such as head and face, nose, mouth and tongue, eye, ear, hands and feet, and others), as well as an overview of important clinical features and recommended diagnostic strategies, could be valuable for professionals in the field of healthcare. This information may be particularly useful for healthcare providers who treat individuals with metabolic disorders or those who care for individuals exhibiting dysmorphic features that could indicate the presence of an inherited metabolic disorder.

## 1. Introduction

Numerous genetic metabolic disorders have dysmorphic features that are part of the condition, highlighting the need for the metabolic geneticist to recognize dysmorphic features and for the general geneticist to know when to consider inborn errors in the differential diagnosis for the patient with dysmorphic features. Communication between and among clinicians depends on consistent terminology in describing these dysmorphic features [1,2]. Standardizing the language used to describe these physical characteristics is crucial for enhancing the accuracy of differential diagnoses and facilitating reliable comparisons among patients [3,4]. In presentation of the dysmorphic features associated with inborn errors of metabolism, we adhered to the consensus guidelines for human malformation terminology outlined in the ‘Elements of Morphology’ website (https://elementsofmorphology.nih.gov/). These guidelines encompass definitions of terms related to various aspects of craniofacial features [5], including the cranium, scalp hair, face, forehead, maxilla and midface, mandible, chin, neck, periorbital region (eyebrow, eye spacing, eyelashes, eyelid, lacrimal puncta) [6], ear [7], nose and philtrum [8], lips, mouth (including the oral region) [9], hands and feet [10].

A child with an inherited metabolic disorder (IMD) may also have another, concurrent condition. The likelihood of this is increased when the child has consanguineous parents, or when the IMD is related to a chromosomal deletion, whether or not the deletion is a recognized contiguous gene syndrome. We know that some IMDs have specific congenital dysmorphic features that are clearly part of the IMD, including Smith-Lemli-Opitz syndrome [11], Zellweger syndrome [12] and some disorders of glycosylation [13]. The occurrence of IMDs is typically higher in regions where consanguineous marriages are common (20–50 %), such as in Middle Eastern countries. This leads to a significant number of rare dysmorphology syndromes [14].

The information provided by this study, including a comprehensive list of IMDs with dysmorphic features stratified according to organs affected (using the categories in the ‘Elements of Morphology: head and face, nose, mouth and tongue, eye, ear, hands and feet, and other) along with an overview of the key clinical features and proposed diagnostic approaches, may benefit any professional in the field and healthcare provider caring for individuals with metabolic conditions, and those caring for individuals with dysmorphic features that should suggest the possibility of IMDs, either individually (e.g., the 2–3 toe syndactyly that suggests Smith-Lemli-Opitz syndrome), as a consequence of another feature of an IMD (e.g., the open mouth that is typical of hypotonia), or more typically, as a constellation of features (e.g., coarse facial features and camptodactyly suggesting a lysosomal storage disorder).

In the Footprints series, the interested reader will find separate publications addressing IMDs that present with specific manifestations of disease or involvement of particular organs or systems, such as cardiac or skeletal malformations. This report focuses on dysmorphic features, most of which are by definition not major malformations; due to the nature of the database, one major malformation (cleft palate) is included in this report, and some examples in our report that illustrate the presence of malformations in IMDs are major malformations.

This resource should not be regarded as a comprehensive list of all IMDs with associated dysmorphic features, as our analysis is based solely on the information currently available in the IEMbase. It is likely that additional disorders fitting this description will be identified in the future.

## 2. Methods

The source of the information was IEMbase, the knowledgebase of IMDs (http://www.iembase.org) [15]. As of November 2024, IEMbase tabulates 1985 disorders and 4464 corresponding clinical and biochemical signs and symptoms grouped in 22 organ and system groups (Autonomic system, Cardiovascular, Dental, Dermatological, Digestive, Dysmorphic, Ear, Endocrine, Eye, Genitourinary, Hair, Hematological, Immunological, Metabolic, Muscular, Neurologic, Psychiatric, Kidney, Respiratory, Skeletal, Tumoral and Other). The clinical symptoms associated with dysmorphisms (*n* = 365) were extracted from the ‘Dysmorphic’ group. The nosology of IMDs [16] was reclassified according to the International Classification of Inherited Metabolic Disorders, ICIMD [17].

## 3. Dysmorphic signs and symptoms

We classified the dysmorphic features of IMDs into seven distinct categories in keeping with the ‘Elements of Morphology”: a) head and face, b) nose and philtrum, c) mouth and tongue, d) periorbital region, e) ear, f) hands and feet, and g) other (Supplemental Table S1). We will focus on highlighting key IMDs that exhibit dysmorphic features and discussing them within the context of their most prominent or frequent abnormalities.

**Fig. 1.**
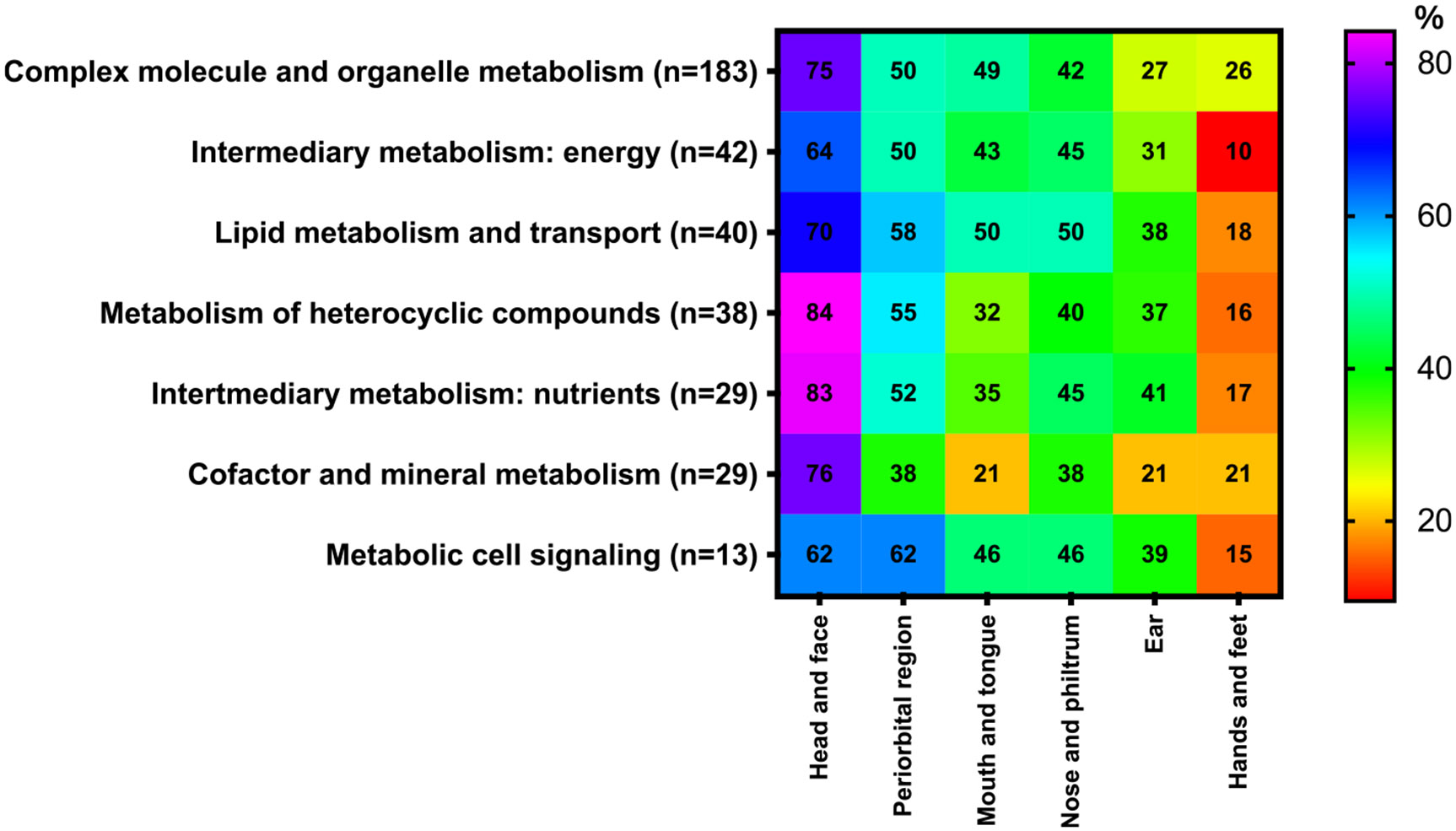
Occurrence (%) of symptoms associated with disorders presenting with dysmorphic phenotype in 7 categories of IMDs. The percentages for dysmorphic abnormalities were calculated using as the denominator the total number of IMDs in each category presenting with any dysmorphic phenotype. Heat scale ranges from red (0 %) for diseases with no particular symptoms reported to violet (100 %) for diseases with particular symptoms occurring with highly frequency within the disorders group. For definition of 6 categories of dysmorphisms see Supplemental Table S2. For interpretation of the references to color in this figure legend, the reader is referred to the web version of this article.

In terms of affected organs, the most commonly reported were dysmorphisms in the head and face in 279 out of 374 (75 %) disorders, followed by abnormalities of the periorbital region in 191 out of 374 (51 %), in the nose and philtrum and mouth and tongue in 161 out of 374 (43 %) each, the ear in 115 out of 374 (31 %), and in the hands and feet in 78 out of 374 (21 %) (refer to Fig. 1 and Supplemental Table S2).

### 3.1. Head and face

Some of the most common IMDs that can present with dysmorphic features in the head and face include Hurler disease (Mucopolysaccharidosis type I; MPS I) or Smith-Lemli-Opitz syndrome. These conditions can manifest with characteristic facial features such as coarse facies, prominent forehead, or other distinctive facial abnormalities.

Among 279 IMDs, the prevalent dysmorphic abnormalities observed in the head and face included micrognathia (13.8 %), coarse face (13.3 %), retrognathia (5.9 %), high forehead (5.7 %), midface hypoplasia (5.7 %) and facial dysmorphism (4.4 %) as detailed in Table 1.

### 3.2. Nose and philtrum

Anomalies of the nose may be classified according to variations in length, width, both length and width, and in shape or position. Some of the most common IMDs that can present with dysmorphic features specifically affecting the nose include Smith-Lemli-Opitz syndrome. These conditions may manifest with characteristic nasal abnormalities such as a flattened nasal bridge, wide nostrils, or other distinctive nasal features.

In 161 IMDs, the common dysmorphic abnormalities noted in the nose and philtrum comprised a broad nasal bridge (14.9 %), depressed nasal bridge (14.1 %), long philtrum (11.2 %), anteverted nares (7.2 %) and short nose (5.6 %) detailed in Table 1.

### 3.3. Mouth and tongue

Some of the most common IMDs that can present with dysmorphic features in the mouth and tongue include Pompe disease, Hurler disease and Fabry disease. These conditions may manifest with characteristic oral and tongue abnormalities such as a high-arched palate, small mouth, macroglossia (enlarged tongue), or other distinctive features.

Among 161 IMDs, the prevalent dysmorphic abnormalities observed in the nose included a high arched palate (15.5 %), cleft palate (12.1 %), thin upper lip (11.6 %) and slender upper lip (6.0 %) as detailed in Table 1.

### 3.4. Periorbital region

The terms used to describe features in the periorbital region include eyebrow, eye spacing, eyelashes, eyelid, and lacrimal punctum. Some of the most common IMDs that can present with dysmorphic features in the eye include: alkaptonuria, Fabry disease, homocystinuria, and mucopolysaccharidoses. These conditions may manifest with characteristic eye abnormalities such as corneal clouding, cataracts, optic nerve atrophy, or other distinctive ocular features.

In 191 IMDs, the common dysmorphic abnormalities noted in the periorbital region consisted of wide-spaced eyes (16.6 %), epicanthal folds (12.5 %), hypertelorism (8.2 %), microphthalmia (6.6 %%), and strabismus (6.0 %) as detailed in Table 1.

### 3.5. Ear

Ear anomalies can involve both quantitative traits and qualitative features of the entire ear, as well as specific components such as variations in size (macrotia, microtia, anotia), position (low-set ears, posterior angulation), and individual anatomical parts like the antihelix, antitragus, concha, helix, lobe, scapha, tragus, or triangular fossa. Some of the most common IMDs that can present with dysmorphic features in the ear include congenital disorders of glycosylation and Smith-Lemli- Opitz syndrome. These conditions may manifest with characteristic ear abnormalities such as malformed or low-set ears or other distinctive features.

In 115 IMDs, the common dysmorphic abnormalities noted in the ear consisted of low set ears (41.2 %), large ears (12.1 %), square ears (8.5 %), posteriorly rotated ears (7.3 %), and dysplastic ears (6.7 %) as detailed in Table 1.

### 3.6. Hands and feet

Many IMDs can manifest with characteristic physical features affecting the hands and feet, such as abnormal finger or toe positioning, joint abnormalities, or other structural anomalies, including the clinodactyly and the 2–3 syndactyly of Smith-Lemli-Opitz syndrome.

Among 78 IMDs, the prevalent dysmorphic abnormalities observed in the hands and feet included clubfoot (16.0 %), syndactyly (8.4 %) and clinodactyly and long digits that each occur in 6.9 % as detailed in Table 1.

### 3.7. Other

Dysmorphic features in other systems are seen in some IMDs. Most geneticists will recognize inverted nipples as an important clue to the potential diagnosis of some congenital disorders of glycosylation, and hypospadias as a feature of Smith-Lemli-Opitz syndrome. Among 102 IMDs, other commonly reported dysmorphic features included short stature (44.1 %), hernias (11.0 %) and inverted nipples and short neck that each occur (9.3 %) and hypospadias (3.4 %) as detailed in Table 1.

## 4. Differential diagnosis

The Supplemental Table S2 provides a detailed compilation of laboratory tests that can assist in diagnosing the different IMDs listed. In cases where metabolite testing does not provide clear results, diagnosis depends on molecular/genetic analysis and a comprehensive clinical history to identify distinctive presenting symptoms. In some conditions for which diagnosis can be made by DNA, biochemical evaluation may be faster or may be required to clarify variations of uncertain significance. However, for about 44 % of the disorders listed, diagnosis can only be confirmed by DNA testing and not by any clinically accessible biochemical testing.

## 5. Conclusions

We have compiled an extensive list of IMDs linked to dysmorphic features and suggested a set of investigations to be conducted based on the organ affected and the treatment options currently available. This marks the 17th installment in a series of educational summaries that offer a thorough and up-to-date compilation of metabolic differential diagnoses categorized by system involvement. The complete list is accessible at www.iembase.org/gamuts and will be regularly maintained and updated. Supplementary data related to this article can be accessed online.

## Supplementary Material

1

2

## Figures and Tables

**Table 1:** Most common dysmorphic features reported in 374 IMDs according to six organ groups. For details also see Supplemental Table S2.

Head and face	%	Nose and philtrum	%	Mouth and tongue	%	Other	%
Micrognathia	13.8	Broad nasal bridge	14.9	High arched palate	15.5	Short stature	44.1
Coarse face	13.3	Depressed nasal bridge	14.1	Cleft palate	12.1	Hernias	11.0
Retrognathia	5.9	Long philtrum	11.2	Thin upper lip	11.6	Inverted nipples	9.3
High forehead	5.7	Anteverted nares	7.2	Slender upper lip	6.0	Short neck	9.3
Midface hypoplasia	5.7	Bulbous nose	5.6	Wide mouth	4.3	Hypospadias	3.4
Facial dysmorphism	4.4	Short nose	5.6	Open mouth	3.4	Retrognathia	2.5
Long face	4.2	Short philtrum	4.4	Small mouth	3.4	Kyphosis	1.7
Frontal bossing	3.7	High nasal bridge	3.6	Thin lips	3.4	Loose, wrinkled skin	1.7
Bitemporal narrowing	3.4	Prominent nose	2.8	Macroglossia	3.0	Pectus carinatum	1.7
Triangular face	3.4	Broad nose	2.4	Tented upper lip	3.0	Short limbs	1.7
Periorbital region	%	Ear	%	Hands and feet	%	Supernumerary nipple	1.7
Widely spaced eyes	16.6	Low set ears	41.2	Clubfoot	16.0	Widely spaced nipples	1.7
Epicanthal fold	12.5	Large ears	12.1	Syndactyly	8.4	Aged appearance	0.8
Hypertelorism	8.2	Square ears	8.5	Clinodactyly	6.9	Barrel-shaped chest	0.8
Microphthalmia	6.6	Posteriorly rotated ears	7.3	Long digits	6.9	Brachycephaly	0.8
Strabismus	6.0	Dysplastic ears	6.7	Camptodactyly	5.3	Broad neck	0.8
Deeply set eyes	5.6	Prominent ears	3.6	Brachydactyly	3.1	Congenital hip dislocation	0.8
Down-slanting palpebral fissures	5.6	Cupped ears	3.0	Tapered digits	3.1	Cryptorchidism	0.8
Up-slanting palpebral fissures	5.3	Large malformed ears	2.4	Adducted thumbs	2.3	Dwarfism	0.8
Synophrys	2.5	Macrotia	2.4	Nail hypoplasia	2.3	Microdontia	0.8
Esotropia	2.2	Large lobes	1.8	Short metacarpals	2.3	Mongolian spot	0.8

## Data Availability

Data will be made available on request.
