# Supplementary material for: Clinical and biochemical footprints of inherited metabolic diseases: XVII. Dysmorphisms
Source: Mol Genet Metab. Author manuscript; Available in PMC 2025 Feb 10. (PMC11808525; doi:10.1016/j.ymgme.2024.109001)
Supplement: 1 [file NIHMS2050528-supplement-1.docx]

**Supplemental Table S1.** Categories of dysmorphic features reported in inherited metabolic diseases.

| **Head and face** |
| --- |
| Aplasia cutis |
| Asymmetric skull |
| Bitemporal narrowing |
| Bony defect of skull |
| Brachycephaly |
| Broad coarse facies |
| Broad forehead |
| Broad forehead with frontal bossing |
| Coarse facies |
| Craniofacial dysmorphia |
| Dolichocephaly |
| Elongated face |
| Facial dysmorphism |
| Facial hirsutism |
| Flat midface |
| Frontal bossing |
| Full cheeks |
| High anterior hairline |
| High forehead |
| Hirsutism |
| Hypoplastic maxilla |
| Infraorbital creases |
| Large fontanelles |
| Long face |
| Low posterior hairline |
| Maxillary prognathism |
| Median facial dysplasia |
| Micrognathia |
| Microretrognathia |
| Midface hypoplasia |
| Myopathic face |
| Narrow forehead |
| Narrow, elongated face |
| Oval face |
| Plagiocephaly |
| Pointed chin |
| Prognathism |
| Prominent cheeks |
| Prominent chin |
| Prominent forehead |
| Prominent forehead and cheekbones |
| Prominent jaw |
| Prominent occiput |
| Receding forehead |
| Retrognathia |
| Round face |
| Saggy cheeks |
| Scaphocephaly |
| Short forehead |
| Short pointed chin |
| Sloping forehead |
| Small triangular face |
| Sparse hair |
| Subtle facial dysmorphia |
| Triangular face |
| Wide anterior fontanel |
| **Nose and philtrum** |
| Anteverted nares |
| Beaked nose |
| Broad nasal bridge |
| Broad nasal root |
| Broad nasal tip |
| Broad nose |
| Bulbous nose |
| Defects of the nose |
| Depressed nasal bridge |
| Flared nostrils |
| Flat nasal root |
| Flat nose |
| High bridge of the nose |
| High nasal bridge |
| Long columella |
| Long nose |
| Long philtrum |
| Long pointed nose |
| Narrow base and nares |
| Nasal hypoplasia |
| Pinched nose |
| Prominent nasal tip |
| Prominent nose |
| Saddle nose |
| Short nose |
| Short nose with prominent nasal tip |
| Smooth philtrum |
| Short philtrum |
| Small nose |
| Square nasal tip |
| Tubular nose |
| Wide nares |
| **Mouth and tongue** |
| Abnormal teeth |
| Bifid incisors |
| Bifid uvula |
| Cleft lip |
| Cleft palate |
| Crowding of the teeth |
| Decayed teeth |
| Downturned mouth |
| Everted lower lip |
| Full lips |
| Full lower lip |
| Gum hypertrophy |
| Gum hypertrophy |
| High arched palate |
| Large mouth |
| Lip papilloma |
| Macroglossia |
| Midline cleft palate |
| Open mouth |
| Pegged teeth |
| Prominent philtrum |
| Protruding tongue |
| Semi-open mouth |
| Short philtrum |
| Short upper lip |
| Small mouth |
| Smooth philtrum |
| Tented mouth |
| Tented upper lip |
| Thick lips |
| Thick upper lip vermilion |
| Thin lips |
| Thin upper lip |
| V-shaped cleft palate |
| Wide mouth |
| **Periorbital region** |
| Wide spaced eyes |
| Almond-shaped eye |
| Amblyopia |
| Arched eyebrows |
| Blepharophimosis |
| Broad and full eyebrows |
| Broad eyebrows |
| Buphthalmus |
| Congenital strabismus |
| Deep-set eyes |
| Down-slanting palpebral fissures |
| Enophthalmos |
| Entropion |
| Epicanthal folds |
| Esotropia |
| Exophthalmus |
| Exotropia |
| High-arched eyebrows |
| Horizontal low-set eyebrows |
| Hypertelorism |
| Hypotelorism |
| Infraorbital puffiness |
| Long eyelashes |
| Long palpebral fissures |
| Medially sparse eyebrows |
| Microphthalmia |
| Narrow palpebral fissures |
| Prominent eyebrows |
| Prominent eyes |
| Proptosis |
| Ptosis |
| Puffy eyelids |
| Round asymmetrical eyes |
| Short palpebral fissures |
| Small orbit |
| Small palpebral fissures |
| Sparse lateral eyebrows |
| Strabismus |
| Synophrys |
| Thick eyebrows |
| Upslanting palpebral fissures |
| **Ear** |
| Cauliflower ear |
| Cupped ears |
| Dysplastic ears |
| Flat ears |
| Fleshy lobes |
| Indented and pointed helices |
| Large ears |
| Large ears with overfolded helices |
| Large lobes |
| Low set ears |
| Macrotia |
| Microtia |
| Posteriorly rotated ears |
| Prominent ears |
| Protruding earlobes |
| Protruding ears |
| Small deep-set ears |
| Square ears |
| Upturned earlobes |
| **Hands and Feet** |
| Sickle foot |
| Abnormal dermatoglyphics |
| Abnormal halluces |
| Abnormal hand morphology |
| Abnormally placed thumbs |
| Absent nails of the fifth digits |
| Adducted thumbs |
| Arthrogryposis |
| Brachydactyly |
| Brachytelephalangy |
| Broad thumbs |
| Camptodactyly |
| Clasped thumb |
| Clinodactyly |
| Clinodactyly of the fifth finger and toe |
| Clinodactyly of the fourth and fifth toes |
| Clinodactyly toes |
| Clubfoot |
| Deformities of the fingers and toes |
| Dorsal foot fat pads |
| Dystrophic nails |
| Fingers, overlapping |
| Flat feet |
| High arches |
| Hindfoot valgus |
| Hyperphalangy of index finger |
| Hypoplastic nails |
| Hypoplastic toe |
| Hypoplastic toenails |
| Large hands |
| Long digits |
| Long fingers |
| Mild camptodactyly |
| Nail hypoplasia |
| Overlapping fingers |
| Overriding toes |
| Pes equinovarus |
| Polydactyly |
| Postaxial polydactyly |
| Proximally placed thumbs |
| Radial deviation of the index finger |
| Rocker bottom feet |
| Short hands and feet |
| Short limbs |
| Short metacarpals |
| Slender digits |
| Slender fingers |
| Slender toes |
| Small feet |
| Small hands |
| Syndactyly |
| Syndactyly of the second and third toes |
| Syndactyly of toes |
| Tapered digits |
| Tapered fingers |
| Terminal transverse defect of toes |
| Thin fingers |
| Thumb hypoplasia |
| Transverse palmar crease |
| Transverse palmar crease |
| Underdeveloped distal phalanges of 3rd fingers |
| **Others** |
| Aged appearance |
| Barrel-shaped chest |
| Broad neck |
| Congenital hip dislocation |
| Cryptorchidism |
| Dwarfism |
| Hypospadias |
| Hypospadias |
| Inverted nipples |
| Kyphosis |
| Laterally facing nipples |
| Loose, wrinkled skin |
| Mongolian spot |
| Narrow chest |
| Pectus carinatum |
| Short limbs |
| Short neck |
| Short stature |
| Supernumerary nipple |
